# Recurrence of Esophageal Residual Gastric Anastomosis With Cervical Lymph Node Metastasis Following Laparoscopic Proximal Gastrectomy for Early-Stage Gastric Cancer

**DOI:** 10.7759/cureus.88730

**Published:** 2025-07-25

**Authors:** Ryo Shibayama, Hayato Shimoyama, Kei Kono, Yutaka Takazawa, Masaki Ueno

**Affiliations:** 1 Department of Gastroenterological Surgery, Toranomon Hospital, Tokyo, JPN; 2 Department of Pathology, Toranomon Hospital, Tokyo, JPN

**Keywords:** 3 fields of lymph node dissection, anastomotic recurrence, esophagectomy, esophagogastric junction, gastric adenocarcinoma, lymphatic metastasis, proximal gastrectomy

## Abstract

Proximal gastrectomy (PG) is performed in Japan for early-stage gastric cancer in the upper stomach using various reconstruction methods. Although the location and frequency of lymph node metastasis in natural esophagogastric junction cancers have been widely discussed, lymph node metastasis of local recurrence to the esophagogastric anastomosis after PG has not been reported. We present the case of a 77-year-old man who underwent laparoscopic PG for early-stage gastric adenocarcinoma. Local recurrence was detected at the esophageal residual gastric anastomosis two years later. Positron emission tomography/computed tomography revealed suspected cervical lymph node metastasis. Intraoperative rapid histopathology confirmed adenocarcinoma, prompting esophagogastrectomy with three-field lymph node dissection.

This report is the first to document cervical lymph node metastasis arising from local recurrence at the esophageal residual gastric anastomosis following PG.

## Introduction

In Japan, proximal gastrectomy (PG) is performed for early-stage gastric cancer located in the upper stomach [1]. In contrast, total gastrectomy is commonly performed in Western countries for gastric cancer. PG prevents postoperative weight loss and malnutrition by preserving gastric function [2]. However, PG has been associated with a higher incidence of anastomosis-related complications, such as anastomotic leakage, stenosis, and reflux symptoms [3]. To mitigate these complications, researchers have developed various reconstructive methods, including esophagogastric anastomosis with an anti-reflux mechanism, double-tract reconstruction, and jejunal interposition reconstruction [4,5].

The frequency of mediastinal lymph node metastasis in esophagogastric junction cancer has been associated with the extent of tumor invasion into the esophagus [6,7]. While these studies have guided resection techniques and the extent of lymph node dissection for esophagogastric junction cancer, no consensus has been reached on an optimal approach. Cervical lymph node metastasis in esophagogastric junction cancer is rare and occurs in approximately 1% of cases [7]. Consequently, cervical lymph node dissection is not routinely performed for this type of cancer.

Although the location and frequency of lymph node metastasis in natural esophagogastric junction cancers have been widely discussed, lymph node metastasis of local recurrence to the esophagogastric anastomosis has not been reported.

Herein, we performed laparoscopic PG for early-stage gastric cancer. Postoperative recurrence occurred at the esophageal residual gastric anastomosis. Positron emission tomography/computed tomography revealed suspected cervical lymph node metastasis. Intraoperative rapid histopathology confirmed adenocarcinoma, prompting esophagogastrectomy with three-field lymph node dissection. To the best of our knowledge, this study is the first to report such findings.

## Case presentation

**Figure 1:**
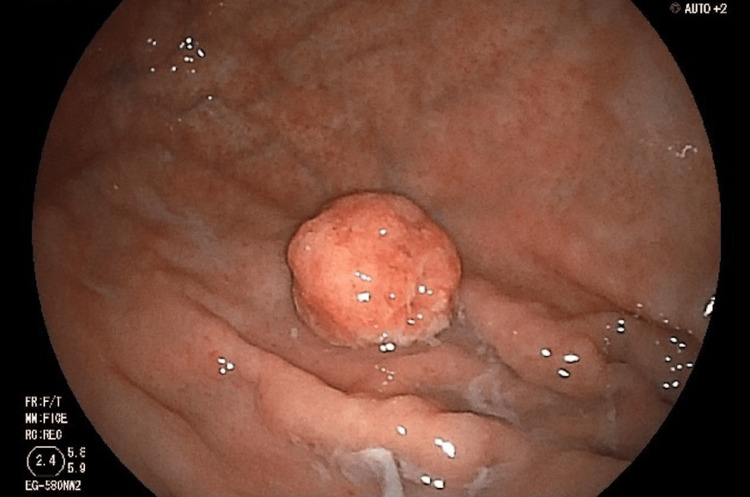
Endoscopic findings of the initial gastric cancer For the primary tumor, esophagogastroduodenoscopy shows mucosal findings of atrophic gastritis in the background and the elevated lesion on the anterior wall of the greater curvature of the fornix of the stomach.

A 77-year-old man was referred to our hospital for esophagogastroduodenoscopy (EGD), which revealed early-stage gastric cancer in the greater curvature of the upper gastric body (Figure 1).

**Figure 2:**
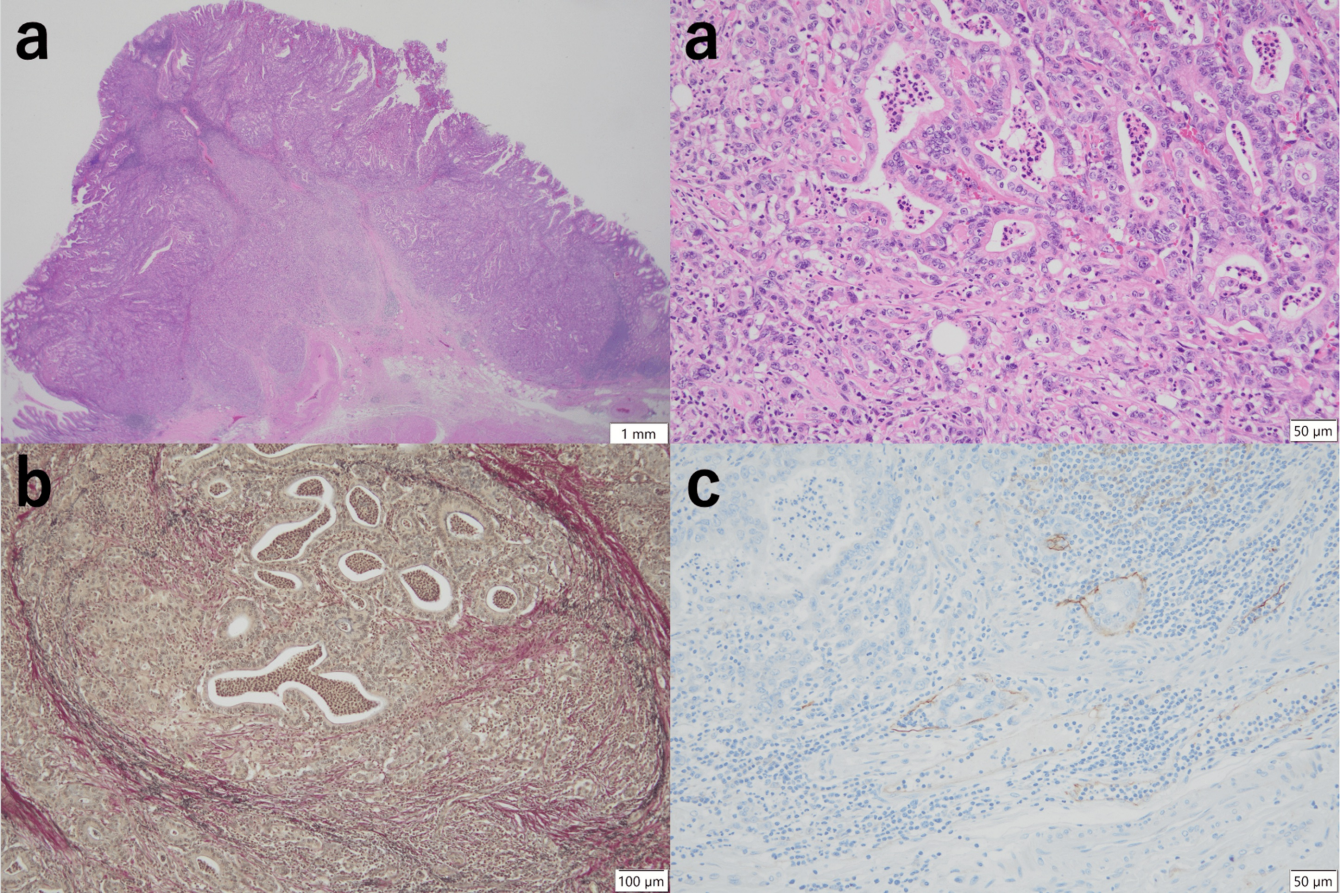
Pathological findings of the PG Histopathological diagnosis of the first surgery, laparoscopic PG, revealed early stomach cancer. The resected specimen measures 10 cm along the lesser curvature and 13 cm along the greater curvature. It is classified as type 1 (mass type) and measures 23 mm × 15 mm × 10 mm, with only a small portion of the esophagus included. Histologically, an 8-mm-thick adenocarcinoma with a predominant tub2 component over por is observed. No invasion of the muscularis propria is observed, with mild lymphatic and moderate venous invasion. No lymph node metastasis is observed. (a) H&E staining at x12.5 (overview) and x200 magnification showing tub2 and por components. (b) Elastic van Gieson staining at x100, highlighting venous invasion. (c) D2-40 staining at x200, indicating lymphatic invasion. PG: proximal gastrectomy; H&E: Hematoxylin and eosin

He underwent laparoscopic PG with modified side overlap with fundoplication using the Yamashita reconstruction method and D1+ lymph node dissection [8]. Histopathological examination confirmed a diagnosis of adenocarcinoma, classified as pT1bN0M0 pStage IA, according to the 8^th^ edition of the UICC staging system. The tumors consisted of moderately to poorly differentiated tubular adenocarcinomas, with evidence of venous and lymphatic invasion. The proximal margin of the tumor was 90 mm, and the distal margin was 27 mm. No tumor cells were found at the resection margins (Figure 2).

**Figure 3:**
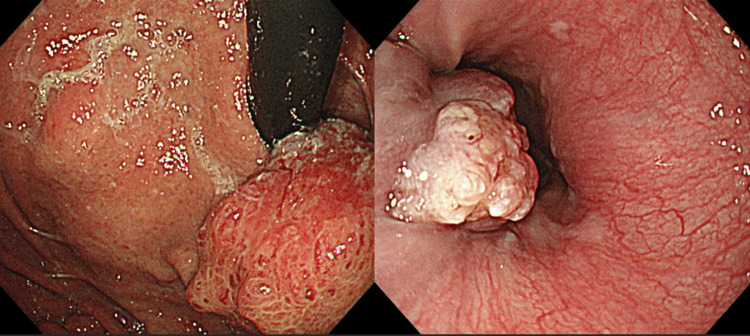
Endoscopic findings of recurrent tumors at the anastomosis site For the second tumor, esophagogastroduodenoscopy revealed a type 1 lesion extending from the residual stomach into the esophagus. Biopsy confirmed adenocarcinoma.

Although this tumor was highly malignant, oncologically complete (R0) resection was achieved. The patient was discharged without postoperative complications and was followed up as an outpatient without additional treatment. No postoperative reflux symptoms were noted, and endoscopic findings showed no evidence of inflammation due to reflux. EGD revealed a recurrent tumor extending from the residual stomach to the esophagus two years after the initial surgery (Figure 3).

**Figure 4:**
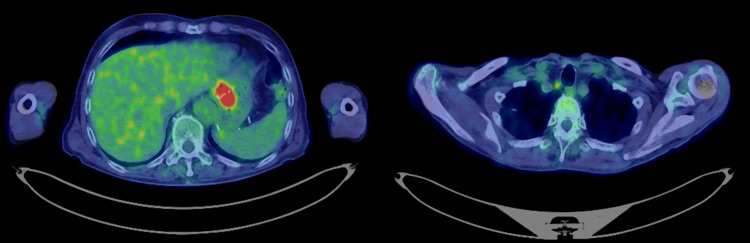
Positron emission tomography/computed tomography Positron emission tomography/computed tomography revealed a primary lesion at the esophagogastric junction and an enlarged right cervical lymph node (no. 101R), both showing significant abnormal fluorodeoxyglucose uptake. No distant metastases were observed.

Biopsy confirmed adenocarcinoma, and positron emission tomography/computed tomography identified a primary lesion at the esophagogastric junction and an enlarged right cervical lymph node (no. 101R) with significant abnormal fluorodeoxyglucose accumulation (Figure 4).

**Figure 5:**
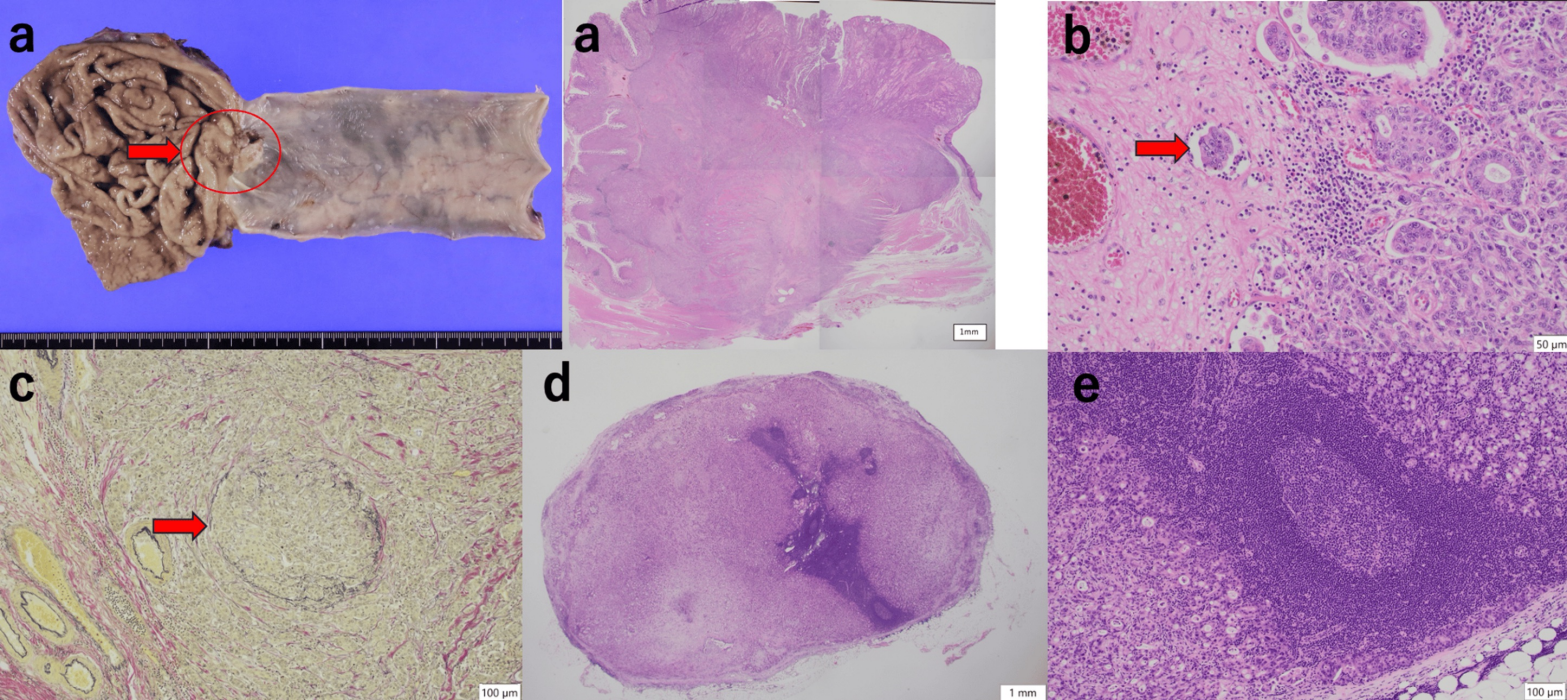
Pathological findings of esophageal resection specimens Histopathological diagnoses of the second surgery, thoracoscopic laparoscopic esophagogastrectomy and the three-field lymph node dissection, are suspected to be local recurrences at the anastomosis. The anastomotic muscularis is the primary site of the moderately to poorly differentiated tubular adenocarcinoma, and no intramucosal carcinoma components are observed. The deepest part of the invasion extends into the subserosal tissue, and lymphatic and venous invasion is observed. The proximal and distal margins are clear. One right cervical lymph node (no. 101R) shows tumor metastasis. H&E staining, x12.5, an overview of the tumor (a). H&E staining, x200, indicating lymphatic invasion (b). Elastic van Gieson staining, x100, highlighting marked venous invasion (c). H&E staining, x12.5, overview of no. 101R (d). H&E staining, x200, showing that tub2 and por components are present in the lymph nodes of no. 101R (e). H&E: Hematoxylin and eosin

These findings suggested local recurrence at the esophageal-residual gastric anastomosis site, with cervical lymph node metastasis (no. 101R). The patient was considered to have metastatic recurrence of gastric cancer, and chemotherapy was considered a viable option for systemic therapy. However, the patient requested radical surgical resection, prompting debate over surgical technique. Sampling dissection of the #101R lymph node was considered; however, the patient preferred a three-field lymph node dissection if the sampling results were positive. The patient then underwent thoracoscopic laparoscopic esophagogastrectomy with three-field lymph node dissection. Intraoperative rapid diagnosis of the right cervical lymph node (no. 101R) confirmed adenocarcinoma. Ileocolic reconstruction was performed via the retrosternal route. Histopathological examination revealed local recurrence at the anastomotic site, with moderately to poorly differentiated tubular adenocarcinoma infiltrating the subserosal tissue. No intramucosal carcinoma components were observed. Esophageal invasion measured 10 mm, with clear proximal and distant margins. Lymphatic and venous invasion was observed, and tumor metastasis was confirmed in one right cervical lymph node (no. 101R) (Figure 5).

Given the similarity of the current specimen to the tumor from the initial surgery, and the presence of tumor invasion primarily in the subserosal tissue, histopathological findings confirmed a local recurrence at the anastomotic region. The postoperative course was uneventful, and the patient was discharged 14 days postoperatively. He survived for 12 months after the esophagectomy without recurrence and was monitored as an outpatient.

## Discussion

There are two possible explanations for the present case. First, the initial gastric cancer independently recurred in the cervical lymph nodes and at the anastomosis site. Alternatively, the cancer may have recurred locally at the anastomotic region, and the recurrent lesion subsequently metastasized to the cervical lymph nodes. Although cervical lymph node metastasis of gastric cancer has been reported, it remains a rare occurrence [8]. Several reports have documented cervical lymph node metastases originating from esophagogastric junction cancers [7,9]. Based on this evidence, the second possibility appears more likely. In the present case, the specimen from the second surgery was determined to originate from anastomotic recurrence rather than a new primary gastric cancer, as the main tumor was located in the muscularis propria and exhibited pathological features similar to those of the original tumor. This tumor was thought to have metastasized to the cervical lymph nodes due to strong lymphatic invasion into the esophageal wall. No reports have documented cervical lymph node metastases arising from local recurrence at the esophageal residual gastric anastomosis after PG for gastric cancer. To our knowledge, this is the first case documenting these findings. Cervical lymph node dissection is not typically performed during surgery for esophagogastric junction cancer. However, the frequency of lymph node metastasis in anastomotic recurrence after PG is expected to be different from that in normal esophagogastric junction cancer. In the present case, the choice of radical resection is considered controversial. However, we opted for esophagogastrectomy with three-field lymph node dissection to achieve R0 resection in this case, and the patient had no recurrence. If double-tract or small bowel interposition reconstruction had been performed, the anastomotic recurrence might have been less likely to metastasize to the mediastinal or cervical lymph nodes, owing to extensive lymphatic drainage to the small intestine [10]. Nevertheless, because cervical lymph node metastasis from esophagogastric junction cancer is rare, the advantages of PG, such as reduced weight loss through gastric preservation and the simplicity of reconstruction with esophageal residual gastric anastomosis, should not be disregarded.

In this case, only one year has passed since the surgery, and long-term outcomes are yet to be determined. We hope that enrolling and examining similar cases in the future will enable us to select the most appropriate treatment strategies.

## Conclusions

This report is the first to document cervical lymph node metastasis arising from local recurrence at the esophageal residual gastric anastomosis following PG. Although there is room for debate regarding the treatment policy, in this case, chemotherapy was not performed, and surgical resection was performed. We hope that this will be helpful in determining treatment plans for similar cases in the future.
